# Learning Curve and Early Results of Interlaminar and Transforaminal Full-Endoscopic Resection of Lumbar Disc Herniations

**DOI:** 10.7759/cureus.7157

**Published:** 2020-03-02

**Authors:** Petr Zelenkov, Vyacheslav V Nazarov, Sergey Kisaryev, Leysan Pimenova, Bahrom A Zakirov, Maria Goldberg, Nikolay A Konovalov, Viktor Feniksov, Aleksei Kondrashov, Ilya Popov, Ruslan I Zagirov

**Affiliations:** 1 Spinal Neurosurgery, N.N. Burdenko National Medical Research Center for Neurosurgery, Moscow, RUS; 2 Neurosurgery, N.N. Burdenko National Medical Research Center for Neurosurgery, Moscow, RUS; 3 Neurosurgery, Gerzen Research Oncological Center, Moscow, RUS; 4 Neurosurgery, Klinikum Muenchen Bogenhausen, Munich, DEU; 5 Neurosurgery, First Moscow State Medical University, Moscow, RUS; 6 Neurosurgery, Moscow Regional Medical Research Center, Moscow, RUS; 7 Internal Medicine, Moscow State University, Moscow, RUS; 8 Neurosurgery, Moscow State Hospital, Moscow, RUS; 9 Radiosurgery, N.N. Burdenko National Medical Research Center for Neurosurgery, Moscow, RUS

**Keywords:** endoscopy, transforaminal, interlaminar, disc herniation, learning curve

## Abstract

Background

Full-endoscopic spinal surgery is an evolving technique. A laborious learning phase is inevitable due to the complexity of the orientation and instrumentation. The goal of the present study is to evaluate a single surgeon’s learning curve and early outcomes in full-endoscopic resection of lumbar disc herniations.

Methods

This was a prospective non-controlled single-surgeon cohort study. In 54 patients with 57 herniations, 41 interlaminar and 16 transforaminal resections were performed. Surgery time, severity of adhesive process in the spinal canal, complication rates and clinical outcomes (VAS, ODI, custom questionnaire, recurrence and re-operation rate) were assessed.

Results

In the interlaminar group, operative time has decreased from 60 ± 20 min in the first 20 operations to 45 ± 14 min in the following 17 (p=0.023). In the transforaminal group, operative time has decreased from 60 ± 16 min in the first 7 operations to 41 ± 12 min in following 9 (p=0.023). Severe adhesive process in spinal canal was associated with duration of symptoms greater than 2 years, longer surgery and higher risk of surgical complications. Four recurrent disc herniations were re-operated using full-endoscopic technique. VAS, ODI and pain medications significantly decreased in both groups and in re-operated patients.

Conclusion

The plateau of the learning curve and good short-term clinical results of full-endoscopic interlaminar and transforaminal surgery may be achieved after twenty operations, given extensive previous experience in microsurgery. Risk of complications at the learning phase may be decreased by excluding the patients with symptoms lasting over two years.

## Introduction

Full-endoscopic lumbar spine surgery via interlaminar (IL) or transforaminal (TF) approach is an evolving technique conquering place from the current “gold standard” - microsurgery [1-9]. Despite the good results and routine use of microsurgery, this method still has several disadvantages such as muscular injury and extensive scar formation within the spinal canal [10]. Full-endoscopic (FE) technique is purported to reduce those consequences.

Based on Nellenstein et al. 2010 systematic review, no advantages of FE over microsurgery technique were demonstrated in terms of clinical outcomes [11]. Nevertheless, further reviews have shown that better clinical outcomes (i.e. shorter recovery period, less postoperative pain) may be achieved with FE compared to microsurgery [2-4]. Evolution of the instrumentation allowed expansion of the indications to FE surgery to any kind of stenosis and revisions in the lumbar spine [1-3,5,6,8,9,12-15].

A laborious training phase is inevitable due to the complexity of the orientation and handling in FE. The discrepancy between proprioception and visual information, absence of depth perception in a two-dimensional monitor view and limited maneuverability of the the instrumentation are the main problems to overcome for surgeons who are accustomed to using a surgical microscope. It remains unclear how much training is needed for mastering the technique.

We found 4 publications and 1 commentary concerning the learning curve in FE lumbar spine surgery [16-20]. Wang et al. reported the learning curve in 30 cases and mean time decrease in the IL approach from 107.9 ± 20.8 in first 10 cases to 43.2 ± 12.7 min in the last 10 [18]. Hsu et al. reported mean time 93.7 ± 44.5 min and no learning curve in 22 IL cases. For TF approach, the same author reported 77.1 ± 43.0 min and a significant learning curve in 34 TF cases [17]. Lee et al. reported significant reduction of TF surgery time after 17 cases, from 62.1 (30-90) to 47.6 (30-105) min [20]. Wang et al. reported TF time of 107 ± 34 min in cases 21 to 40 for the surgeon inexperienced in FE and 70 ± 13 min for another surgeon with 2 years of “demonstration teaching” [19]. The best overall results for FE surgery time were reported by Ruetten et al. 2008: mean time 22 (13-46) min for all FE procedures with 13-46 min range for IL and 14-37 min for TF [7]. Low rate of complications was shown by all the authors and no conclusive analysis could be performed due to small sample sizes.

The goal of the present study is to assess a single surgeon’s (first author) learning curve and early clinical outcomes in full-endoscopic interlaminar and transforaminal removal of lumbar disc herniations.

## Materials and methods

Study design

Prospective non-controlled single-surgeon case series.

Patient characteristics

The study population consisted of patients with symptomatic lumbar disc herniation that presented to the first author between February 2013 and March 2015. Patients were enrolled prospectively on intent-to-treat basis. All patients signed written informed consent for the procedure.

Inclusion criteria

Confirmation of diagnosis was made by the surgeon based on 1.5 tesla MRI dated less than 3 month prior to admission, with T2-weighted sagittal and axial series. Surgical indications included radicular pain resistant to conservative treatment over at least 4 weeks and/or motor or sensory deficit associated with the affected nerve root [21,22]. Disc herniations at one or two levels between L3 and S1 were included. Any localization (central, lateral, foraminal) and any sequester orientation was accepted as suitable for full-endoscopic approach, either IL or TF. All cases were evaluated and treated by the first author.

Exclusion criteria

Patients were excluded if they had undergone previous lumbar surgery or or had a history of relevant trauma/tumor/infection, unstable spondylolisthesis, severe stenosis or scoliosis affecting the index level. There were no limitations due to medical comorbidities, unless they proved contraindications to surgery. Any body mass index (BMI) was accepted, unless it was surgically prohibitive (e.g. operational table).

Ethical approval

All patient care and procedures performed in the study were in accordance with the ethical standards of the institutional and national research committee and with the 1964 Helsinki declaration and its later amendments or comparable ethical standards.

Surgical procedure

All operations were performed by a single surgeon (first author), with 7 years’ experience in spinal microsurgery. Prior to the study, the surgeon attended a basic course of full-endoscopic lumbar surgery at Richard Wolf training center in St. Anna’s hospital, Herne, Germany. Training included lectures and cadaver hands-on practice. After first 17 cases, he attended an advanced course at the same facility.

Operations were done under general endotracheal anesthesia, in prone position with hips and knees flexed. Two-plane radiological control was used for level localization and intraoperative instruments positioning.

At L5-S1, procedures were performed exclusively via posterior interlaminar (IL) approach. At L4-L5 and L3-L4 lateral transforaminal (TF) approach was indicated, unless the foramen was overlaid by the iliac crest beyond the cranial pedicle, as specified by Ruetten et al [6].

The surgical technique of IL and lateral TF approaches strictly corresponded to those described in detail by Ruetten et al 2007 [6]. Vertebris (Richard Wolf) endoscopic system was used, with 7.9 mm working sheath, 205*6.9 mm rod lens optics with 4.2 mm working channel and corresponding instrumentation. Constant irrigation with saline under hydrostatic pressure of 3 m was applied. Bipolar coagulation with 300 kHz was applied using the specially designed probe, Trigger-Flex (Richard Wolf) [6].

The extent of removal of herniated material, neural decompression and mobility were estimated visually, by palpation with surgical instruments and by intermittent increase of saline pressure (flow test) to visualize the mobility of contents in the spinal canal. In the TF approach, due to limited the visualization of the spinal canal, only the flow test (see methods) and estimation of the total amount of material removed were used as relevant signs of sufficient decompression.

The skin incision, which did not exceed 8 mm, was sutured intracutaneously with 1-2 resorbable stitches.

Postoperatively, all patients were prescribed oral non-steroidal anti-inflammatory medication (predominantly Meloxicam 15 mg) daily for a period of 5 days. Patients were mobilized on the first day after surgery.

Intraoperative findings

Surgery time was recorded from incision to skin closure. Learning curves for IL and TF approach were assessed separately as a function of surgery time relative to the number of corresponding procedures performed.

Intraoperatively, extent of adhesions/scarring in spinal canal around the neural structures was recorded in terms of “no adhesions”, “light”, “moderate” and “severe” according to surgeon’s judgement assisted by a custom assessment chart, Table 1.

**Table 1:** Custom assessment chart for adhesions in full-endoscopic spinal surgery.

Sign/ Severity	Epidural fat	Epidural veins	Adhesions	Dural wall visualization
No adhesions	Bulky	Normal	No	Reduced by bulky fat
Light	Normal with few adhesions	Normal or hypertrophic	Few	Easy by sliding the fat apart
Moderate	Atrophic, partially replaced by adhesions	Hypertrophic	Partially covers the contents of the canal	Demands mobilization of fat and adhesions by pulling
Severe	Trace, replaced by adhesions	Hypertrophic or replaced by adhesions	Fully covers the contents of the canal	Demands dissection and coagulation of adhesions

Outcome parameters

Intraoperative and postoperative complications, re-herniations and re-operations were recorded. Postoperative or follow-up MRI was not performed on regular basis, unless it was clinically indicated.

Leg pain and back pain duration prior to surgery were recorded. VAS (visual analog scale) for leg pain and back pain, ODI (Oswestry disability index) (validated Russian translation of version 2.1a [23]), neurology, pain therapy and employment data was collected pre-operatively and at the day of discharge (except for ODI). Follow-up data was collected via phone call at average term of 11 month (± 7 month SD). All patients were asked to report their back and leg pain in 0-10 score (auditory analogue of VAS) and to respond to ODI questionnaire and custom questionnaire presented in Table 2. 

**Table 2:** Custom phone-call questionnaire.

Question	Response
Pain medication (regardless of the type used)	Never – 0, Less than once a week – 1, Once a week – 2, Each second day – 3, Daily – 4, Several times a day – 5
Unemployment	No, Yes (due to spine-related reasons), Yes (due to other reasons)
Numbness in lower limb	Yes-1, No-0
Burning sensation in lower limb	
Weakness in lower limb	
Inability to rise on toe	
Foot drop/stepping gait	
Pain at bed rest	
Any urination disturbances	
Satisfaction with surgery	
Acceptance of repeated full-endoscopic surgery, if necessary	

Statistical analysis

All data was gathered and processed in Google Spreadsheets. For sample description in quantitative variables, average and standard deviations were taken for normal distributions variables, and medians, 1st and 3rd quartiles for non-normal distributions. Mann-Whitney test was used to estimate the differences and Spearman test for correlations using www.socscistatistics.com service. A statistical significance was stated if the p≤0,05. For estimation of trends, polynomial trendlines and R2 were calculated by Google Spreadsheets function.

## Results

Patient population

Total number of 54 patients were enrolled (25 females and 29 males). The age (average 45 ± 14 years), body mass index (BMI, average 28 ± 8 ), comorbidities, employment, smoking and sports involvement were evenly distributed in the population.

**Figure 1:**
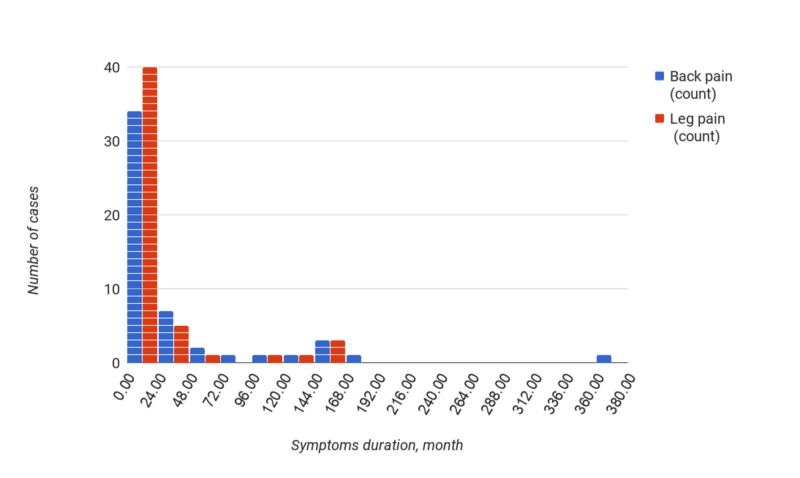
Duration of back and leg pain prior to surgery, in months.

Median duration of symptoms prior to surgery was 4 months (quartiles 2 and 16 months) for leg pain, and 11 months (quartiles 4,5 and 33 months) for back pain (Figure 1).

There were no cases of discrepancy between the MRI and clinical symptoms. Fifty-seven herniations were diagnosed in 54 patients (21 right- and 36 left-sided). Double-level clinically symptomatic herniations were found in 3 patients.

Baseline clinical parameters of patients are presented in Table 3.

**Table 3:** Baseline data and clinical outcomes for IL, TF, double operations and reoperations. In brackets medians (1st, 3rd quartiles) are shown. Scoring for pain medication was as follows: Never – 0; Less than once a week – 1; Once a week – 2; Each second day – 3; Daily – 4; Several times a day – 5

	Groups	Patients with single IL operation	Patients with single TF operation	Patients with double operations	Reoperated patients
Parameters	N of cases	35	10	3	6
VAS leg	preop	8 (7, 10)	10 (8.7, 10)	8 (7.2, 8)	9 (8, 10)
	postop	1 (0, 2.75)	4 (2.5, 7)	2 (0.5, 5)	4 (3, 6)
	FU	1 (0, 1)	3 (1, 3)	1,5 (0, 3)	1 (0.7, 1,7)
VAS back	preop	7 (3.5, 8)	2 (2, 6,75)	5 (1.2, 6,5)	7 (2, 8)
	postop	1 (0, 2)	2 (1.7, 2.2)	5 (1.2, 5,7)	2 (2, 5)
	FU	0 (0, 2)	1 (1, 1,5)	0.5 (0, 1)	1.5 (0.7, 2)
ODI	preop	60 (44, 70)	54 (48, 64.5)	44 (44, 47)	85 (70, 90)
	FU	4 (2, 14)	9 (2.5, 14)	8 (2, 14)	15 (14, 16)
Pain medication	preop	4 (2.5, 5)	3.5 (3, 4)	4 (3.2, 4.7)	4 (4, 4)
	FU	0 (0, 0.5)	1 (0.2, 1.7)	0 (0, 1.5)	1 (0.2, 1)
Sensory deficit, N of patients	preop	10	5	3	3
	postop	17	7	3	5
	FU	10	7	3	5
Foot drop/stepping gait	postop	1	0	0	1
Satisfaction with surgery, N of patients	FU	33	8	2	3
Acceptance of repeated full-endoscopic surgery, if necessary, N of patients	FU	31	6	2	3

Surgical procedure

Fifty-seven full-endoscopic procedures were performed and 3 patients underwent 2-level operations. Distribution of approaches and levels is demonstrated in Table 4.

**Table 4:** Distribution of surgical approaches and levels.

	Interlaminar	Transforaminal
L5-S1	36	0
L4-L5	3	12
L3-L4	2	4
Total	41	16

Intraoperative findings

No bone resection was needed in both IL and TF approach. At L3-L4 and L4-L5, patient positioning was sufficient to open the interlaminar window for IL approach. Forty-four herniations were non-contained while 13 were contained. Total removal of herniated material was possible in all TF and in all but one IL cases. In this case, removal was stopped due to dural rupture and neural damage. This case is described in complications section. Sequester removal in one piece was possible in 13 cases, among them in 2 TF cases with large sequester migration beyond the middle of the pedicle (at L4-L5 and L3-L4). The disc space was accessible and debulked in 25 of 41 IL cases and all TF cases. Bipolar coagulation was not always sufficient to control bleeding, which diminished visualization. In those cases, temporary increase of saline pressure enabled the surgeon to visualize the source of bleeding and coagulate it.

Adhesions

According to our classification of adhesive process (see Methods), “light” adhesions were found in 36 levels, “moderate” in 14 levels and “severe” in seven levels. No patients were adhesion-free in terms of our classification.

There was a strong correlation between the adhesion severity and duration of symptoms (Spearman test, R=0,815, p<0,001). Among 10 patients with duration of leg symptoms longer than 24 month, adhesions were “moderate” in four and “severe” in seven herniations. Vice versa, “severe” adhesive process was found only in patients with duration of symptoms above 24 month (7 cases).

Herniation calcification was found in six cases. It was accompanied by “moderate” adhesions in three cases and “severe” in another three cases. All patients had suffered leg symptoms longer than 24 month.

Surgery time

**Figure 2:**
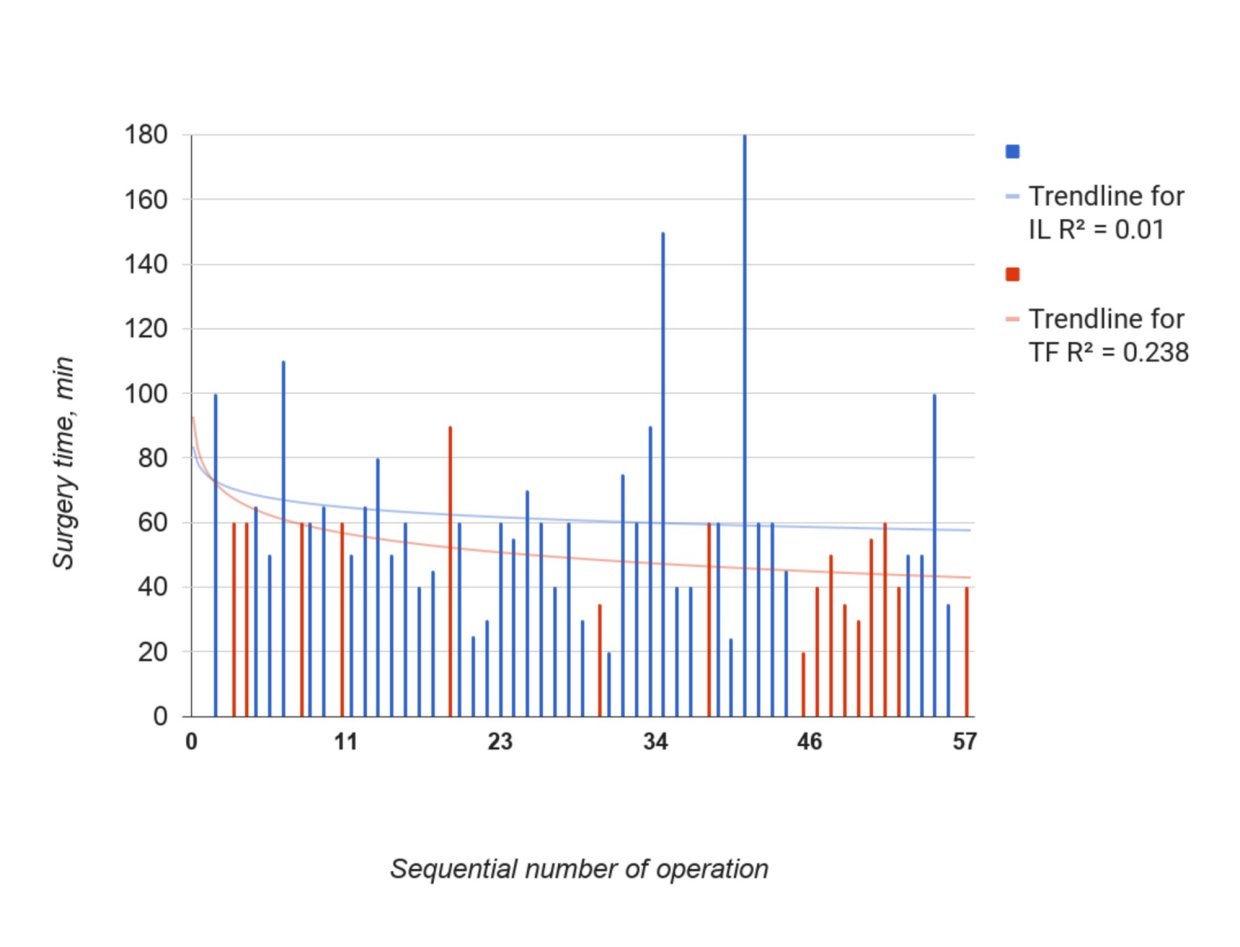
Surgery time in min for IL (blue) and TF (red) groups. Order 3 polynomial trendlines and R2 were calculated by Google Spreadsheet function.

Figure 2 demonstrates dynamics of surgery time throughout the series.

In the TF group, the trendline showed constant relevant decrease of surgery time after 7 cases. In the IL group, there was a weak fit of the trendline to data (R^2^=0.107). The trend increased in second part of the series due to remarkably outlying surgery time in operations N34, 40 and 54. In all three cases, “severe” adhesive process was present, and major complications occurred and had to be dealed with in two of them (N29 and N41). The Spearman test revealed strong positive correlation of surgery time with adhesion severity (R=0.473, p=0,001) and moderate positive correlation with duration of leg pain (R= 0.311, p=0,020).

**Figure 3:**
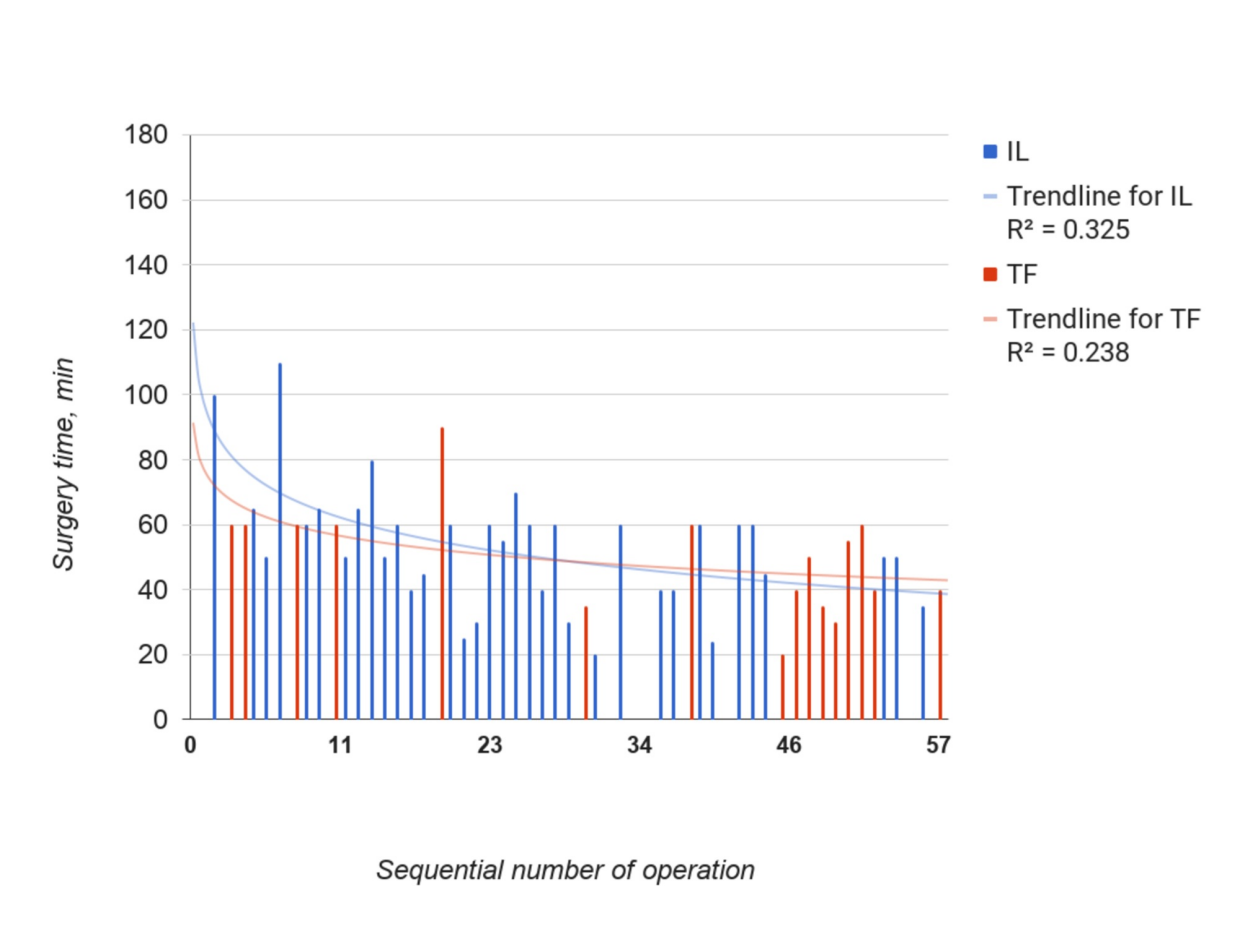
Surgery time in min for IL (blue) and TF (red) groups excluding "severe" adhesion cases. Order 3(IL) and 5(TF) polynomial trendlines and R2 were calculated by Google Spreadsheet function.

In order to eliminate the influence of adhesive process on learning curve, additional analysis was performed excluding “severe” adhesions cases (Figure 3).

The trendline for IL came to the plateau at about 20th case. When comparing the first 20 and following 17 cases, surgery time was significantly lower in later group: average 45 ± 14 (SD) min vs. 60 ± 20 (SD) min (p=0.023). Spearman test revealed weak negative correlation between the surgery time and the number of procedures performed (p=0.007).

Similarly, based on the curve form, the TF group was divided into first seven and later nine cases. Surgery time was significantly lower in the later group: average 41 ± 12 (SD) min vs. 60 ± 16 (SD) min (p=0.012). Spearman test revealed weak negative correlation between the surgery time and the number of procedures performed (p=0.0057).

There was a very weak positive Pearson correlation between surgery time and BMI (p=0.038), not confirmed by Spearmen test (p=0.28).

Complications

Blood loss was not measurable in all cases. There were no postoperative hematomas, wound infections, deep vein thrombosis or urinary retention. There were no conversions to open surgery in primary cases.

Dural ruptures occurred in 4 IL operations (N1, 26, 29, 41). In case N1, it was due to excessive pushing of sleeve into the spinal canal at the approach. Acute motor and sensory deficit was present postoperatively, possibly due to compression of nerve roots by the sleeve. Full-endoscopic revision was undertaken; it was converted to microsurgery to enable dural repair. No additional decompression was found to be necessary and no additional herniation material was obtained. At the follow-up, neurological deficit was partially recovered and ODI decreased to 14 (from 92 preoperatively).

In cases N26, 29 and 41, “severe” adhesions were present. In N26 and N29 neural fascicles (2-3) were damaged and a persistent neurological deficit was present at the follow-up in case N29. In case N41, no neural damage occurred, but psychomotor agitation was seen at awakening. Lumbar puncture revealed intradural bleeding, which was successfully treated by saline washing and intravenous tranexamic acid. No deficit occurred in this patient and she was pain-free at the follow-up. No conversions, dural repair attempts or reoperations were needed in these 3 patients and no cerebrospinal fluid leaks or pseudomeningoceles were observed postoperatively.

Neuritis, described as a burning sensation in the leg, occurred in three patients; all recovered after 1-3 month of conservative treatment. In these patients, no “severe” adhesions were found.

Neurological outcomes are presented in Table 3.

Recurrences and reoperations

There were six repeat surgeries, all using same full-endoscopic technique as in primary surgery. First re-operation is described above. The second was done in case N2 due to persistent radicular pain five days after primary surgery; additional herniated material was removed that was possibly overlooked at the primary surgery.

The remaining four re-operations were done due to recurrent disc herniations confirmed by MRI: two in the IL group (both within first two months) and two in TF group (one at two weeks and the other in two month).

Clinical outcomes

None of the patients was lost to follow-up. In all types of surgery (single IL or TF operation, double operations and re-operations), VAS, ODI and pain medication scores diminished significantly from the preoperative to the postoperative stage as well as to the follow-up (p<0,0001) (Table 4). No differences in those parameters between the groups were found during the follow-up. Median values for ODI, VAS, pain medications and frequencies of selected parameters are shown in Table 4. There were no urinary disturbances, pain at bed rest, or S1 motor deficit preoperatively or postoperatively. Postoperative dysesthesia was a common in all groups. Two patients were unemployed due to spinal reasons prior to surgery and at the follow-up. In two patients, persistent foot drop was present at the follow-up, one of which existed preoperatively.

The vast majority of IL and TF patients were satisfied with the operation and they would consider repeated full-endoscopic surgery if indicated. Among re-operated patients, half were satisfied.

## Discussion

In our series, surgery time for the IL approach decreased after 20 cases to a plateau in 17 cases at 45 ± 14 (SD) min, and there was a negative correlation between the time of surgery and number of operations done. This finding is in line with Wang et al who reported mean time 43 ± 12 min for IL surgery after 20 cases [18].

For the TF approach, time seemed to stabilize at 41 ± 12 (SD) min after 7 cases and there was also a negative correlation between the time of surgery and number of operations. That result corresponds to Lee et al, who reported 47 (30-105) min in TF cases 18 to 34 [20].

It remains unclear to us whether the surgical learning curves may be defined as “steep” or “shallow” according to Benzel et al [16]. Furthermore, given the small size of our series, they still may represent fluctuations at the top of the learning curve.

Both IL and TF techniques utilize the same instrumentation and are very similar in handling. In present series, both procedures were evenly distributed within the time scale, probably providing “cross-training” effect. Moreover, besides the series described, during the timeframe of the study, the first author performed 17 other types of FE interventions using the Vertebris system: two thoracic herniations via posterolateral approach, one TF lumbar microdiscectomy relapse, one lumbar synovial cyst, five single-level IL lumbar stenosis, three TF lumbar stenosis, four carpal and one cubital tunnel decompressions.

The presence of an adhesive process in the spinal canal is viewed differently in FE approach than under the microscope. This may be attributed to better visualization due to water constantly washing the blood away, better illumination and closer inspection of structures of interest. High incidence and morphological features of inflammatory adhesions accompanying disc herniation were described by Ruetten et al [6]. To our knowledge, the presented custom scale for grading the adhesions severity in FE surgery is the first published. According to our results, severe adhesions in the spinal canal, predictable by duration of symptoms over 2 years, significantly increase surgery time and the risk of surgical complications.

Given the calcification of herniations observed in patients with leg symptoms present over two years, preoperative CT scan may be reasonable for those patients. Nevertheless, in several patients with long-lasting symptoms, adhesions were “light” or “moderate” and surgery time was similar with adhesion-free cases.

Sequester migration beyond the middle of the pedicle was described as a contraindication for TF approach by Ruetten et al [6]. In our series, we performed TF approach in two such cases in an attempt to remove the sequester in one piece, which was successfully achieved.

Neurological outcomes in all groups were reasonable, including patients with re-operations. High discrepancy of follow-up term (± 7 month) could lead to bias in estimation of postoperative status, although the data obtained was evenly distributed.

## Conclusions

The plateau of learning curve and good short-term clinical results of full-endoscopic interlaminar and transforaminal surgery may be achieved after the first twenty operations, given extensive previous experience in microsurgery. Risk of complications at the learning phase may be decreased by excluding the patients with symptoms lasting over two years.
